# Supplementary material for: In vivo screening identifies GATAD2B as a metastasis driver in KRAS-driven lung cancer
Source: Nat Commun. 2018 Jul 16;9:2732. doi: 10.1038/s41467-018-04572-3 (PMC6048166; doi:10.1038/s41467-018-04572-3)

Supplementary Information

for

***In Vivo* Screening Identifies *GATAD2B* as a Metastasis Driver in *Kras*-Driven Lung Cancer**

Caitlin Grzeskowiak[1,*], Samrat T. Kundu[2,*], Xiulei Mo[3], Andrei A. Ivanov[3], Oksana Zagorodna[1], Hengyu Lu[1], Richard H. Chapple[1], Yiu Huen Tsang[1], Daniela Moreno[1], Maribel Mosqueda[4], Karina Eterovic[4], Jared J. Fradette[2], Sumreen Ahmad[2], Fengju Chen[5], Zechen Chong[6], Ken Chen[6], Chad J. Creighton[5,6,8], Haian Fu[3], Gordon B. Mills[4], Don L. Gibbons[2,7,#], Kenneth L. Scott[1,#]

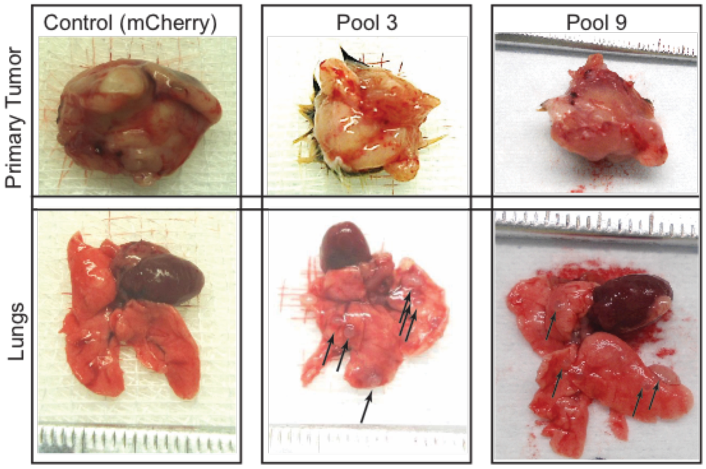

Supplementary Figure 1. In vivo tumor growth by 393P cells (top row): 393P murine tumor cells form subcutaneous tumors in immune competent mice in both pooled cell lines and GFP. In metastatic tissues (bottom row), lungs represent metastatic events in the pooled cell lines.

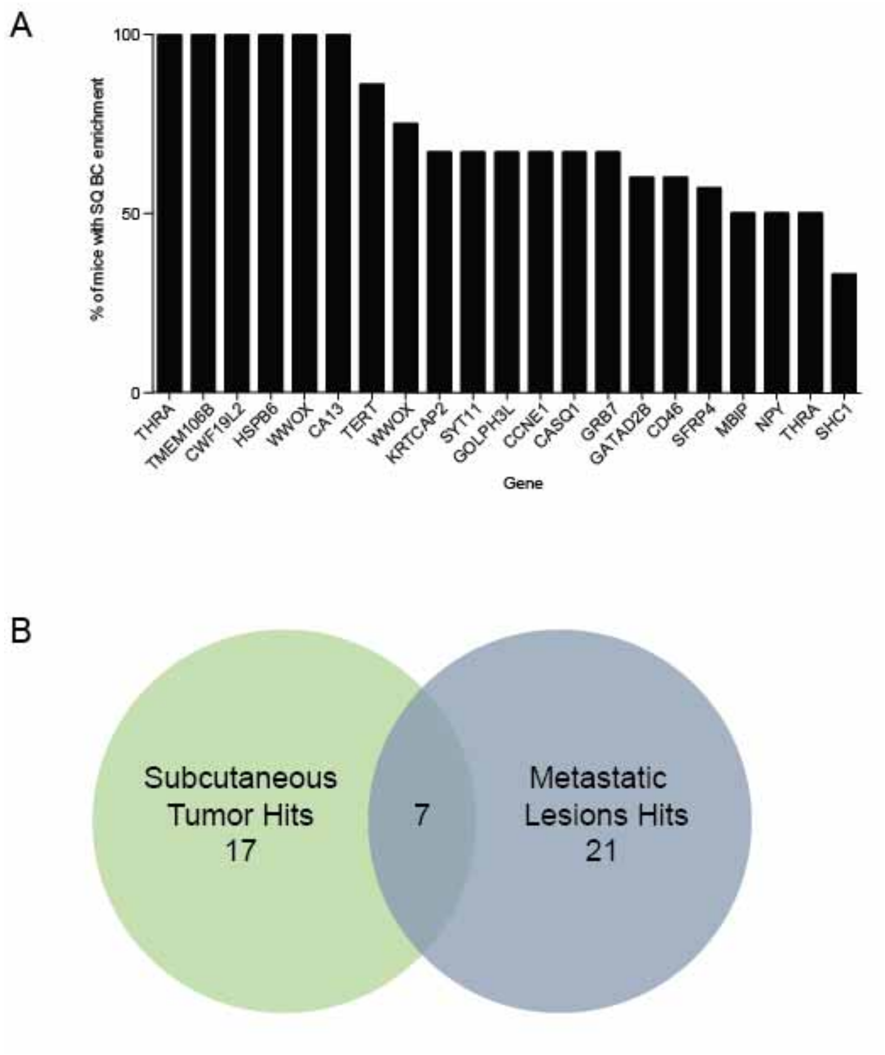

Supplementary Figure 2. Barcode screening results reveal unique and overlapping tumor progression drivers (A) Genes identified in subcutaneous tumors with barcode enrichment having > 100% enrichment over input. Bars represent percent of mouse cohort with >100% for individual ORFs (21 ORFS, 19 unique genes). Duplicate ORFs resulted from screening of multiple ORFs for certain genes when available in our ORF collection **(B)** Barcode sequencing from screen identified 17 ORFs with enrichment only in subcutaneous tumors, 21 ORFs only in metastatic lesions, and 7 ORFs where the barcode was found to be enriched in both primary tumor and metastatic lesions.

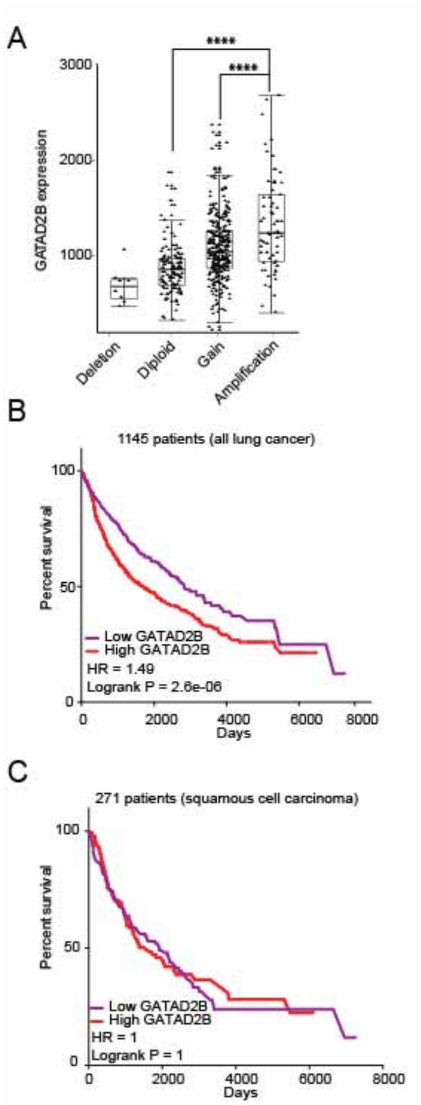

Supplementary Figure 3. *GATAD2B* expression and patient survival (A) *GATAD2B* RNA expression correlates with putative GISTIC copy number amplification (n=125 diploid, n=312 gain, n=65, ****p<0.0001). **(B)** Increased *GATAD2B* expression correlates with worse patient prognosis in analysis of across 1,145 lung cancer patients regardless of molecular subtype (Gyorffy et al., 2013). **(C)** *GATAD2B* expression does not correlate with expression in a subset of lung cancer: squamous cell carcinoma (Gyorffy et al., 2013).

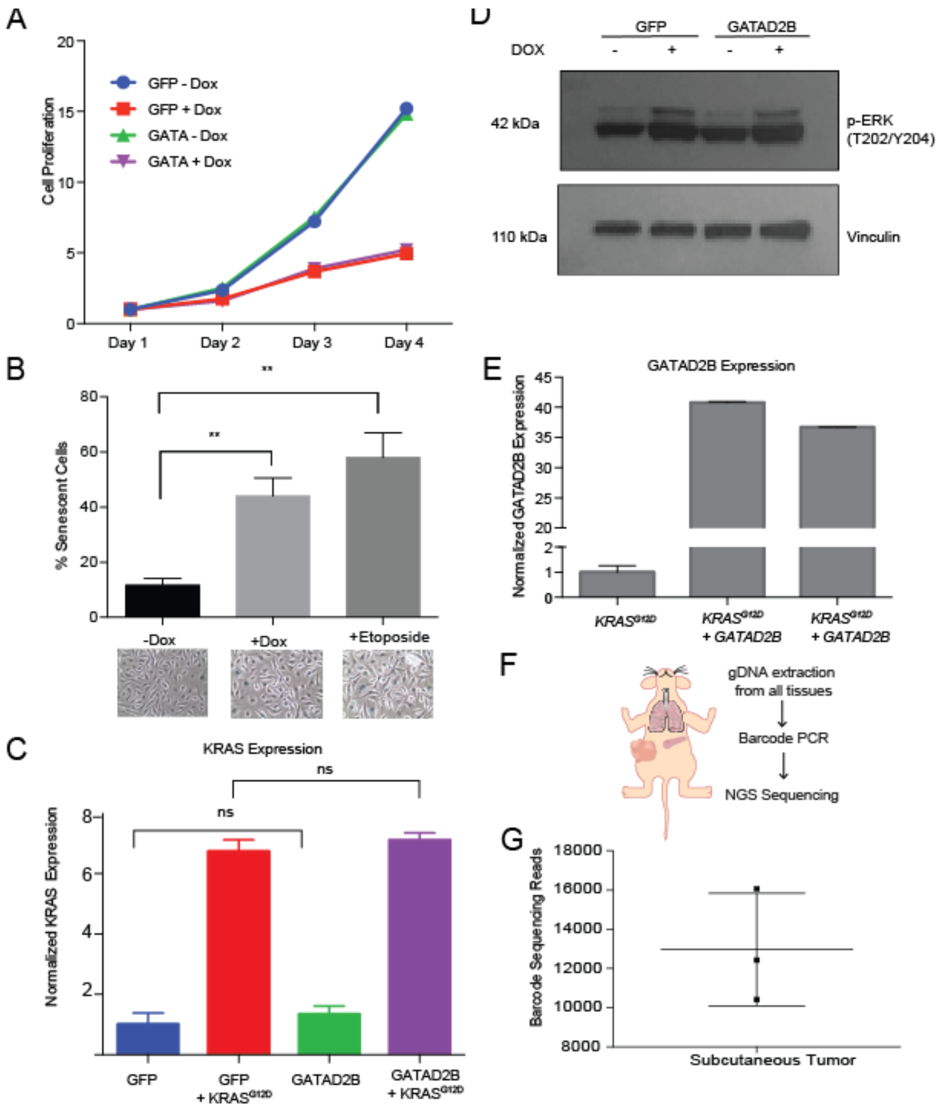

Supplementary Figure 4. HBEC as a model for *KRAS* effectors (A) *GATAD2B* does not significantly change proliferation *in vitro* compared to GFP (n=4). **(B)** *KRAS*^{G12D} (Dox) induces senescence in HBEC cells **(C)** Exogenous *GATAD2B* does not significantly alter *KRAS* expression levels compared to GFP (n=3), error bars represent s.e.m. **(D)** *GATAD2B* does not alter downstream *KRAS* signaling pathway MAPK (assessed by use of anti-phospho-ERK$^{T202/Y204}$ antibody) in HBECs. **(E)** *GATAD2B* gene expression of subcutaneous tumors (2 shown) driven by GATAD2B and GFP (n=3). **(F)** Schematic map

demonstrating metastatic sites where *GATAD2B*-Barcode was present. Elements of image used with permission of Patrick J. Lynch and Carl Jaffe, MD under Creative Commons Attribution 2.5 License 2006 **(G)** Barcode reads present in *GATAD2B*-driven subcutaneous tumors.

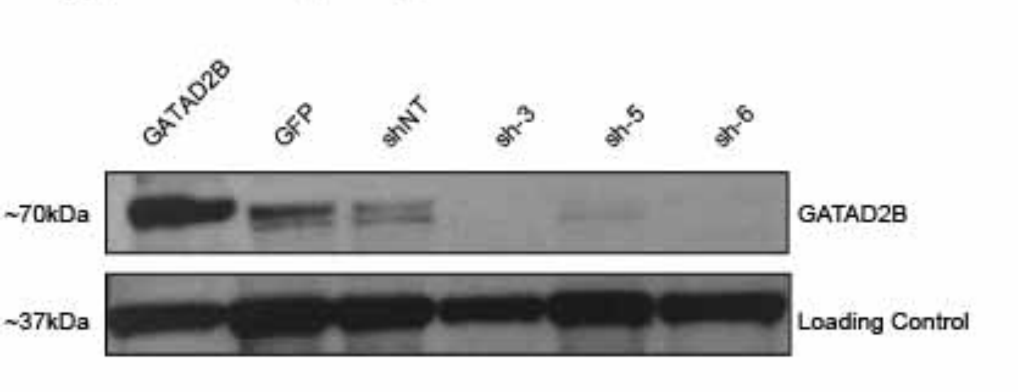

Supplementary Figure 5. Knockdown of *GATAD2B* in NSCLC cell lines (A) Representative immunoblot of *GATAD2B* knockdown with shRNA vs shNT, GFP and *GATAD2B* overexpression.

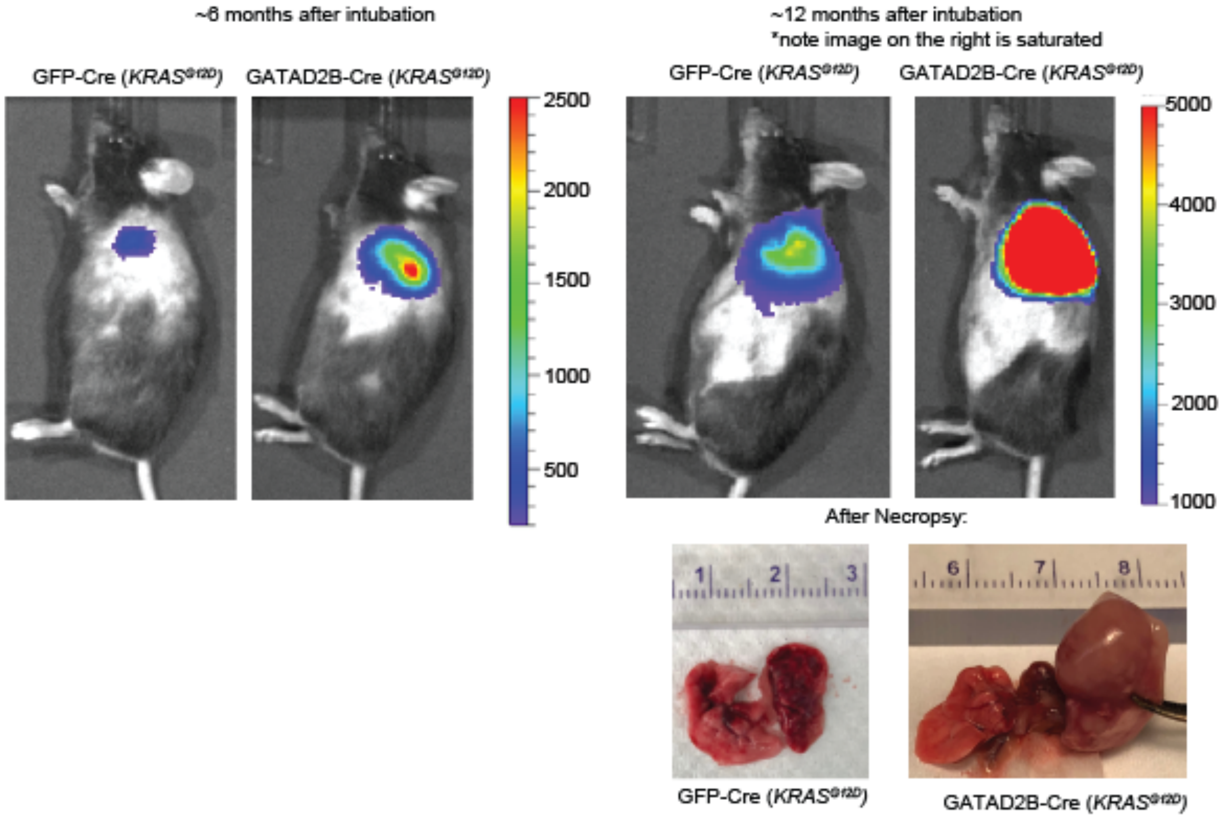

Supplementary Figure 6. Serial Imaging monitors enhanced tumor growth Luciferase signal tracks relative tumor growth in GATAD2B and GFP-cre treated mice.

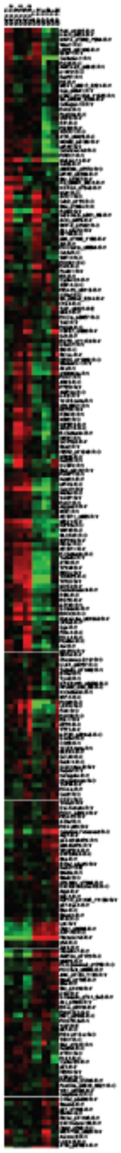

Supplementary Figure 7 Pathway signaling altered in GATAD2B-driven tumors. Protein changes measured across 247 antibodies via RPPA in *GATAD2B* vs. *GFP*- driven tumors (unsupervised clustering).

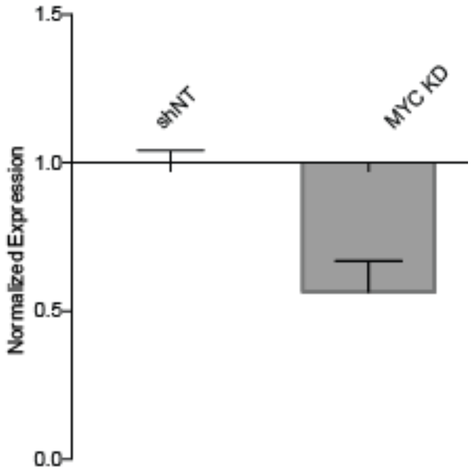

Supplementary Figure 8 MYC knockdown efficiency in HBECs. Gene expression analysis via qPCR of *MYC* reveals lower *MYC* expression relative to non-targeting vector. error bars represent s.e.m.

Supplementary Table 1 Necropsy Report

Pool #	ORFS Injected	Mice Injected	Mice Euthanised	Mice alive with tumors	Mice with Metastasis	Mice with no metastasis
1	19+Mcherry	10	10	0	2	8
2	19+Mcherry	10	10	0	2	8
3	19+Mcherry	10	10	0	8	2
4	19+Mcherry	10	10	0	8	2
5	19+Mcherry	10	10	0	3	7
6	19+Mcherry	10	10	0	2	8
7	19+Mcherry	10	10	0	9	1
8	19+Mcherry	10	9	0	6	3
9	19+Mcherry	10	10	0	9	1
10	19+Mcherry	10	10	0	6	4
11	19+Mcherry	10	10	0	9	1
12	9+Mcherry	10	8	0	7	1
Total	218	120	117	0	71	46

Supplementary Table 2 Top Hits from in vivo Screen TCGA KRAS correlation

	Survival correlation (compendium n=1492)		GISTIC amplification (firehose, n=515)	% gain (~3+ copies)	% gain (~5+ copies)	Correlation DNA copy v expression (n=512 adenos)			Significance of mutation, pan-cancer	diff Ex, kras mut vs wildtype (TCGA, n=230)	
Cytoband	beta (>0, correlation with worse outcome)	p-value	TCGA Amp_genes .conf_99 (wide peak)	TCGA (n=515)		r-value (Pearson's)	t-statistic (Pearson's)	P-value	in top 260 list from Lawrence et al Nature 2014	ttest	fold (kras mut/wt)
12q13.13	-0.049869132	0.211999175	---	4.4%	1.2%	0.282205294	6.643108364	7.89433E-11	yes	0.000697994	higher_mut
8q22.2	-0.13895044	0.000757126	---	22.5%	9.5%	0.581359942	16.13594608	1.27083E-47	---	0.416642668	---
8q12.3	0.024551636	0.547652658	---	19.0%	7.2%	0.091397279	2.0727165	0.038700404	---	0.573657569	---
19q12	0.116197024	0.067688717	yes	7.2%	4.1%	0.796911226	29.79125251	1.0239E-113	---	0.562003716	---
1q43	0.072690843	0.236737368	---	20.6%	5.0%	0.004404705	0.099473208	0.920801647	---	0.395337003	---
7p22.3	-0.046039384	0.467651527	---	17.9%	6.8%	0.509347155	13.3664858	3.77766E-35	---	0.702372793	---
11q13.3	0.025598232	0.534865744	---	7.0%	2.1%	0.289292082	6.824964562	2.50379E-11	---	0.45611943	---
11q22.3	0.179852259	0.006169768	---	3.8%	1.2%	0.558709895	15.21341494	2.28415E-43	---	0.023134311	higher_wt
1q23.3	0.024363152	0.547620026	---	25.0%	8.2%	-0.143333895	-3.27070714	0.00114533	---	0.494911452	---
4q31.3	-0.102495647	0.020539528	---	1.5%	1.0%	0.275172026	6.463794015	2.39048E-10	yes	0.547593023	---
1q21.3	-0.072967168	0.247957702	yes	29.3%	11.8%	0.514618474	13.55430378	5.79013E-36	---	0.005962235	higher_mut
20q13.32	-0.037584581	0.356149808	---	12.4%	5.6%	0.630986339	18.36786424	3.29083E-58	---	0.002778813	higher_wt
17q24.3	0.000187814	0.996225773	---	9.3%	2.9%	-0.07184888	-1.626780524	0.104401378	---	0.653730464	---
14q13.3	-0.19596666	0.00000413	yes	20.0%	13.0%	0.418034083	10.39213238	4.48527E-23	---	0.586797046	---
20q13.12	0.206954292	0.000000232	---	7.2%	2.1%	0.35405751	8.549553172	1.44555E-16	---	0.005128671	higher_wt
8q24.21	0.098748012	0.016224898	yes	27.2%	13.4%	0.276066886	6.486545424	2.0798E-10	---	0.367189577	---

7p15.3	0.039032348	0.325036064	---	17.3%	6.8%	0.138194643	3.151109059	0.001722079	---	0.004087429	higher_wt
15q25.3	-0.029003478	0.445593207	---	1.5%	0.2%	0.011061968	0.249829695	0.802819651	---	0.75105174	---
17q12	-0.129439883	0.045159245	yes	7.2%	2.1%	0.303043774	7.181384846	2.45679E-12	---	0.971284905	---
1q23.2	-0.003811472	0.925267529	---	25.9%	8.9%	0.587760738	16.40659491	6.92506E-49	---	0.2839634	---
7p21.3	-0.078790932	0.059043375	---	17.9%	7.0%	0.04833166	1.092759619	0.275015262	---	0.157282605	---
1q24.2	-0.12335267	0.002858784	---	22.3%	6.6%	0.386415008	9.461393034	1.11434E-19	---	0.663013292	---
1q21.3	0.121134148	0.052764362	yes	29.3%	11.7%	0.493575405	12.81644643	8.53472E-33	---	0.188746901	---
1q21.3	0.028202371	0.51752755	yes	29.1%	12.6%	0.603263527	17.08199683	4.57362E-52	---	0.595584869	---
1q21.3	0.007522334	0.905048847	yes	29.0%	12.4%	0.509819341	13.38322616	3.1977E-35	---	0.487570678	---
17q21.1	-0.057813399	0.14249273	---	6.7%	1.9%	0.194928984	4.488212016	8.88595E-06	---	0.441255464	---
7p21.3	0.048481725	0.259684337	---	18.3%	7.2%	0.496349081	12.91192618	3.35775E-33	---	0.00977718	higher_wt
16q23.1	-0.052211465	0.185681931	---	3.4%	1.2%	0.425571936	10.62051741	6.13106E-24	---	0.122255062	---
1q32.1	-0.0585717	0.155410523	---	22.5%	5.8%	0.522339228	13.83318623	3.49676E-37	---	0.00011231	higher_mut

Supplementary Table 3 Hallmark pathways upregulated in GATAD2B-KRAS HBECs

Overlap Results

Collection(s):	H
# overlaps shown:	10
# genesets in collections:	50
# genes in comparison (n):	426
# genes in universe (N):	45956

Gene Set Name	# Genes in Gene Set (K)	Description	# Genes in Overlap (k)	k/K	p-value	FDR q-value
HALLMARK_G2M_CHECKPOINT	200	Genes involved in the G2/M checkpoint, as in progression through the cell division cycle.	23	0.115	1.87E-18	9.37E-17
HALLMARK_E2F_TARGETS	200	Genes encoding cell cycle related targets of E2F transcription factors.	21	0.105	3.77E-16	9.43E-15
HALLMARK_EPITHELIAL_MESENCHYMAL_TRANSITION	200	Genes defining epithelial-mesenchymal transition, as in wound healing, fibrosis and metastasis.	18	0.09	7.12E-13	1.19E-11
HALLMARK_MYC_TARGETS_V2	58	A subgroup of genes regulated by MYC - version 2 (v2).	11	0.1897	5.88E-12	7.35E-11
HALLMARK_MTORC1_SIGNALING	200	Genes up-regulated through activation of mTORC1 complex.	17	0.085	7.82E-12	7.82E-11
HALLMARK_ESTROGEN_RESPONSE_EARLY	200	Genes defining early response to estrogen.	14	0.07	6.92E-09	5.77E-08
HALLMARK_DNA_REPAIR	150	Genes involved in DNA repair.	11	0.0733	1.79E-07	1.28E-06

HALLMARK_UV_RESPONSE_UP	158	Genes up-regulated in response to ultraviolet (UV) radiation.	11	0.0696	3.03E-07	1.90E-06
HALLMARK_APOPTOSIS	161	Genes mediating programmed cell death (apoptosis) by activation of caspases.	11	0.0683	3.66E-07	2.04E-06
HALLMARK_ESTROGEN_RESPONSE_LATE	200	Genes defining late response to estrogen.	12	0.06	4.40E-07	2.20E-06

Supplementary Table 4 Enriched pathways identified via RPPA

Overlap Results

Collection(s): C1, C2, C3, C4, C5, C6, C7, H
overlaps shown: 100
genesets in collections: 13311
genes in comparison (n): 47
genes in universe (N): 45956

Gene Set Name	# Genes in Gene Set (K)	Description	# Genes in Overlap (k)	k/K	p-value	FDR q-value
PID_MTOR_4PATHWAY	69	mTOR signaling pathway	7	0.1014	7.54E-13	2.01E-09
GRESHOCK_CANCER_COPY_NUMBER_UP	323	Genes from common genomic gains observed in a meta analyis of copy number alterations across a panel of different cancer cell lines and tumor samples.	10	0.031	1.05E-12	2.34E-09
SEMENZA_HIF1_TARGETS	36	Genes that are transcriptionally regulated by HIF1A [GeneID=3091].	6	0.1667	1.56E-12	2.60E-09
PID_MET_PATHWAY	80	Signaling events mediated by Hepatocyte Growth Factor Receptor (c-Met)	7	0.0875	2.20E-12	3.26E-09
PID_DELTA_NP63_PATHWAY	47	Validated transcriptional targets of deltaNp63 isoforms	6	0.1277	8.54E-12	1.14E-08
SIGNAL_TRANSDUCTION	1634	Genes annotated by the GO term GO:0007165. The cascade of processes by which a signal interacts with a receptor, causing a change in the level or activity of a second messenger or other downstream target, and ultimately effecting a change in the functioning of the cell.	15	0.0092	4.43E-11	4.21E-08

PID_P53_DOWNSTREAM_PATHWAY	137	Direct p53 effectors	7	0.0511	1.02E-10	7.74E-08
PID_IGF1_PATHWAY	30	IGF1 pathway	5	0.1667	1.26E-10	8.36E-08
HARRIS_HYPOXIA	81	Genes known to be induced by hypoxia	6	0.0741	2.52E-10	1.59E-07
MARTINEZ_TP53_TARGETS_DN	593	Genes down-regulated in mice with skin specific knockout of TP53 [GeneID=7157].	10	0.0169	4.00E-10	2.29E-07
PROTEIN_COMPLEX	816	Genes annotated by the GO term GO:0043234. Any protein group composed of two or more subunits, which may or may not be identical. Protein complexes may have other associated non-protein prosthetic groups, such as nucleic acids, metal ions or carbohydrate groups.	11	0.0135	5.04E-10	2.48E-07
DANG_BOUND_BY_MYC	1103	Genes whose promoters are bound by MYC [GeneID=4609], according to MYC Target Gene Database.	12	0.0109	8.30E-10	3.95E-07
SHIPP_DLBCL_VS_FOLLICULAR_LYMPHOMA_UP	45	Top 50 up-regulated markers distinguishing diffuse large B-cell lymphoma (DLBCL) from follicular lymphoma (FL) samples.	5	0.1111	1.06E-09	4.57E-07
GSE39110_DAY3_VS_DAY6_POST_IMMUNIZATION_CD8_TCELL_WITH_IL2_TREATMENT_UP	199	Genes up-regulated in CD8 T cells after immunization: day 3 versus day 6 and IL2 [GeneID=3558] treatment.	7	0.0352	1.39E-09	5.70E-07
FARMER_BREAST_CANCER_APOCRINE_VS_LUMINAL	326	Genes which best discriminate between two groups of breast cancer according to the status of ESR1 and AR [GeneID=2099;367]: apocrine (ESR1- AR+) and luminal (ESR1+ AR+).	8	0.0245	1.45E-09	5.70E-07

SWEET_LUNG_CANCER_KRAS_UP	491	Genes up-regulated in the Kras2LA mouse lung cancer model with mutated KRAS [GeneID=3845].	9	0.0183	1.60E-09	6.10E-07
PHOSPHOTRANSFERASE_ACTIVITY_ALCOHOL_GROUP_AS_ACCEPTOR	334	Genes annotated by the GO term GO:0016773. Catalysis of the transfer of a phosphorus-containing group from one compound (donor) to an alcohol group (acceptor).	8	0.024	1.76E-09	6.51E-07
MACROMOLECULAR_COMPLEX	945	Genes annotated by the GO term GO:0032991. A stable assembly of two or more macromolecules, i.e. proteins, nucleic acids, carbohydrates or lipids, in which the constituent parts function together.	11	0.0116	2.33E-09	7.94E-07
BENPORATH_MYC_TARGETS_WITH_EBOX	230	Set 'Myc targets1': targets of c-Myc [GeneID=4609] identified by ChIP on chip in cultured cell lines, focusing on E-box-containing genes; high affinity bound subset	7	0.0304	3.81E-09	1.17E-06
KINASE_ACTIVITY	369	Genes annotated by the GO term GO:0016301. Catalysis of the transfer of a phosphate group, usually from ATP, to a substrate molecule.	8	0.0217	3.83E-09	1.17E-06
PID_SHP2_PATHWAY	58	SHP2 signaling	5	0.0862	3.95E-09	1.17E-06
KRIEG_HYPOXIA_NOT_VIA_KDM3A	770	Genes induced under hypoxia independently of KDM3A [GeneID=55818] in RCC4 cells (renal carcinoma) expressing VHL [GeneID=7428].	10	0.013	4.87E-09	1.38E-06
WINTER_HYPOXIA_METAGENE	242	Genes regulated by hypoxia, based on literature searches.	7	0.0289	5.41E-09	1.50E-06
PUJANA_BRCA1_PCC_NETWORK	1652	Genes constituting the BRCA1-PCC network of transcripts whose expression positively correlated (Pearson correlation	13	0.0079	7.18E-09	1.87E-06

		coefficient, PCC >= 0.4) with that of BRCA1 [GeneID=672] across a compendium of normal tissues.				
PID_HIF1_TFPATHWAY	66	HIF-1-alpha transcription factor network	5	0.0758	7.66E-09	1.96E-06
MARTINEZ_RB1_AND_TP53_TARGETS_DN	591	Genes down-regulated in mice with skin specific double knockout of both RB1 and TP53 [GeneID=5925;7157] by Cre-lox.	9	0.0152	7.99E-09	2.01E-06
PID_VEGFR1_2_PATHWAY	69	Signaling events mediated by VEGFR1 and VEGFR2	5	0.0725	9.61E-09	2.25E-06
TRANSFERASE_ACTIVITY_TRANSFERRING_PHOSPHORUS_CONTAINING_GROUPS	424	Genes annotated by the GO term GO:0016772. Catalysis of the transfer of a phosphorus-containing group from one compound (donor) to another (acceptor).	8	0.0189	1.13E-08	2.46E-06
GSE21360_SECONDARY_VS_QUATERNARY_MEMORY_CD8_TCELL_DN	162	Genes down-regulated in memory CD8 T cells: 2' versus 4'.	6	0.037	1.67E-08	3.52E-06
BYSTRYKH_HEMATOPOIESIS_STEM_CELL_QTL_TRANS	882	Transcripts in hematopoietic stem cells (HSC) which are trans-regulated (i.e., modulated by a QTL (quantitative trait locus) not in a close proximity to the gene).	10	0.0113	1.76E-08	3.65E-06
PID_MYC_ACTIV_PATHWAY	79	Validated targets of C-MYC transcriptional activation	5	0.0633	1.91E-08	3.78E-06

Supplementary Uncropped Western Blots:

Figure 4E:

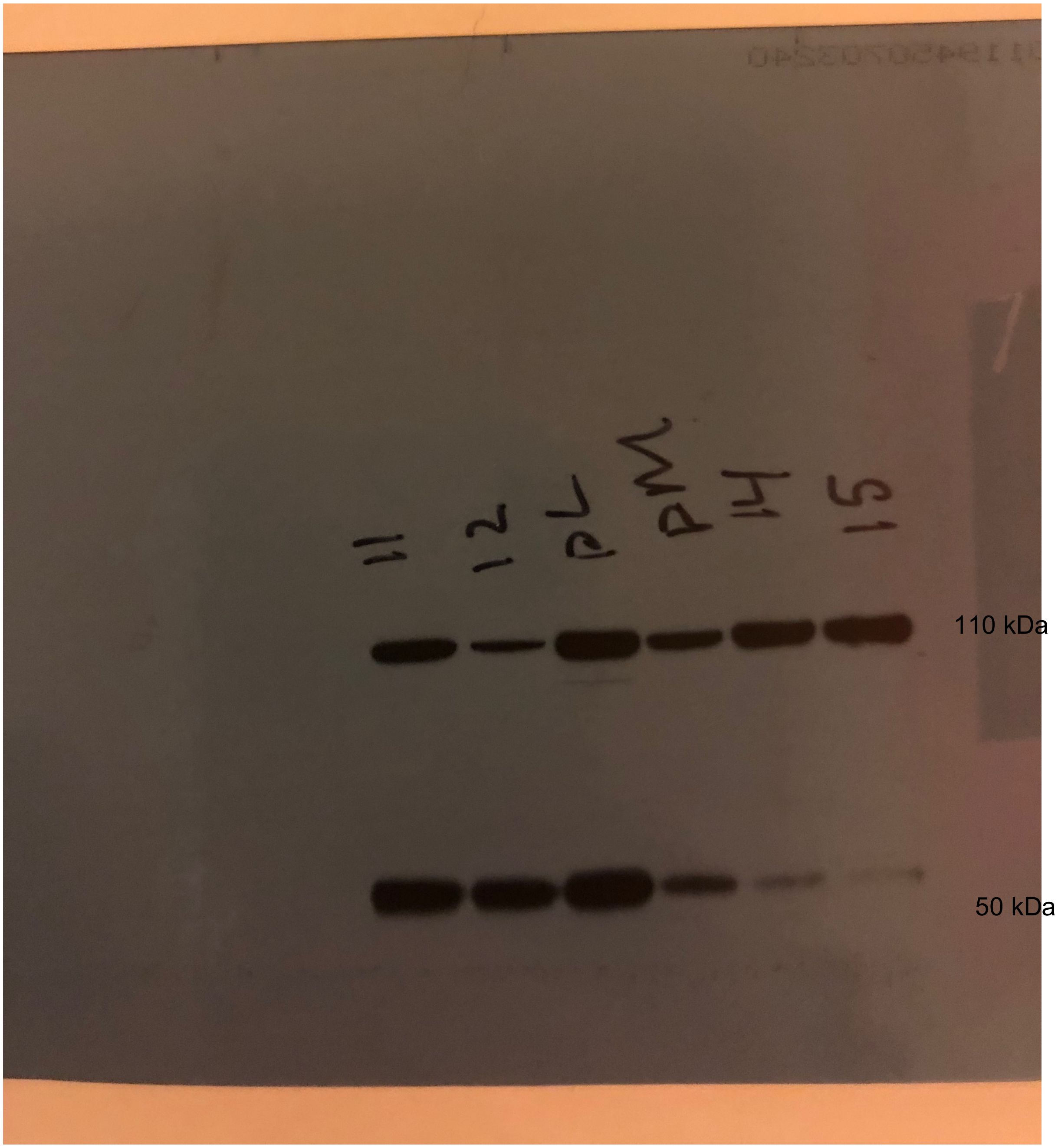

Figure 4I:

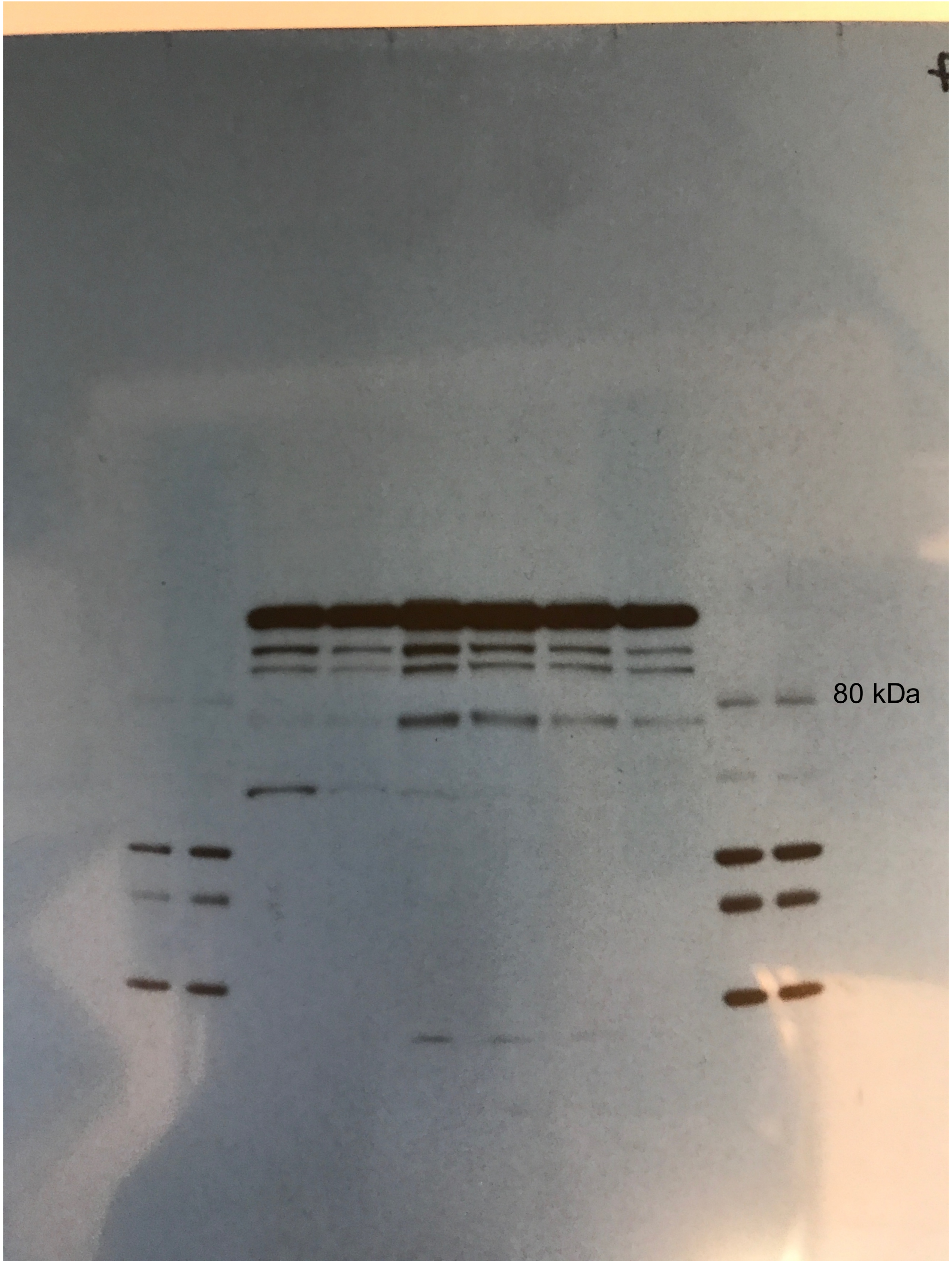

Supplement: Supplementary file 1 — Supplementary Information [file 41467_2018_4572_MOESM1_ESM.pdf]
