# Supplementary material for: In vivo screening identifies GATAD2B as a metastasis driver in KRAS-driven lung cancer
Source: Nat Commun. 2018 Jul 16;9:2732. doi: 10.1038/s41467-018-04572-3 (PMC6048166; doi:10.1038/s41467-018-04572-3)
Supplement: Supplementary file 3 — Description of Additional Supplementary Files [file 41467_2018_4572_MOESM3_ESM.pdf]

Description of Additional Supplementary Files

File Name: Supplementary Data 1
Description: Gene Candidate List Generation. Tab 1: List of ORFs generated from *in silico* analysis of murine models detailing those available to us identified by the bioinformatic interrogation, but were not used for technical purpose as described. Tab 2: Resulting analysis of comparing gene expression profiles of $KRAS^{G12D}$ and $KRAS^{G12D};p53^{R172H\Delta G}$ mice. Tab 3: Conservation analysis to compare homology of genes selected for screen.

File Name: Supplementary Data 2
Description: Information on isoforms used in this screen, including 24 nucleotide DNA barcode for each gene. Tab 2: Raw barcode read counts results from all samples analyzed. Tab 3: Individual gene enrichment assessment for subcutaneous and metastatic sites.

File Name: Supplementary Data 3
Description: Microarray analysis of HBECs +/- mutant *KRAS* and +/- *GATAD2B.*

File Name: Supplementary Data 4
Description: Comparison of previously published Ras signaling signatures to the Dox-treated HBEC-*iKRASG12D* cells.
